# Identifying musical pieces from fMRI data using encoding and decoding models

**DOI:** 10.1038/s41598-018-20732-3

**Published:** 2018-02-02

**Authors:** Sebastian Hoefle, Annerose Engel, Rodrigo Basilio, Vinoo Alluri, Petri Toiviainen, Maurício Cagy, Jorge Moll

**Affiliations:** 1 Cognitive and Behavioral Neuroscience Unit and Neuroinformatics Workgroup, D’Or Institute for Research and Education (IDOR), Rio de Janeiro, Brazil; 2 Biomedical Engineering Program, COPPE, Federal University of Rio de Janeiro, Rio de Janeiro, Brazil; 3 Day Clinic for Cognitive Neurology, University Hospital Leipzig, Leipzig, Germany; 4 Max Planck Institute for Human Cognitive and Brain Sciences, Leipzig, Germany; 5 Finnish Centre for Interdisciplinary Music Research, Department of Music, Art and Culture Studies, University of Jyväskylä, Jyväskylä, Finland; 6 International Institute of Information Technology, Gachibowli, Hyderabad, India

## Abstract

Encoding models can reveal and decode neural representations in the visual and semantic domains. However, a thorough understanding of how distributed information in auditory cortices and temporal evolution of music contribute to model performance is still lacking in the musical domain. We measured fMRI responses during naturalistic music listening and constructed a two-stage approach that first mapped musical features in auditory cortices and then decoded novel musical pieces. We then probed the influence of stimuli duration (number of time points) and spatial extent (number of voxels) on decoding accuracy. Our approach revealed a linear increase in accuracy with duration and a point of optimal model performance for the spatial extent. We further showed that Shannon entropy is a driving factor, boosting accuracy up to 95% for music with highest information content. These findings provide key insights for future decoding and reconstruction algorithms and open new venues for possible clinical applications.

## Introduction

Encoding and decoding models were first introduced in the visual and semantic domains^1–4^. These previous studies established both the theoretical and practical advantages of using rich stimulus descriptions for analysing functional magnetic resonance imaging (fMRI) data. Encoding models enable the explicit assessment of different theoretic models or representations in the brain. For example, using the same visual stimulus set and brain data, the anterior occipital cortex was better modelled by a semantic model as compared to a visual model that in contrast showed highest accuracies in early visual cortices^5^. A decoding stage may be used in combination with encoding, adding practical applications such as the so-called “brain reading” approach^6^. These brain reading or decoding approaches attempt to make inverse inferences from brain activity to the stimulus space. Two main applications can be distinguished, namely identification and reconstruction. Identification aims to identify a specific stimulus from a finite stimulus dataset. Reconstruction aims to build a “replica” of the original stimulus based on a learned mapping between stimulus features and brain activity.

In analogy to the visual and semantic studies that employed natural movies and spatiotemporal Gabor wavelet models^2^, musical stimuli can be described in temporal and frequency dimensions. In contrast to auditory models for spectro-temporal receptive fields, which are used for encoding natural sounds such as animal cries and environmental sounds^7^, here we employ musical features comprising low-level as well as higher-level characteristics of musical dimensions (tonality, dynamics, rhythm, timbre^8^) and that have been thoroughly validated in behavioural studies of music perception^9–11^. Such musical features have been explored using computational models to investigate brain responses to naturalistic musical stimuli using fMRI^12–14^. However, these studies have either focused on encoding models of musical/acoustic components^12,13^ or on the direct decoding of these components from brain activity^14^. The combination of encoding and decoding models in the musical domain has the potential to extend our comprehension of how complex musical information is structured in the human auditory cortex. This will foster the development of models that can ultimately decode music from brain activity, and may open the possibility of reconstructing the contents of auditory imagination, inner speech and auditory hallucinations^15^. Similarly, brain-computer interfaces for locked-in syndrome patients^16,17^ could be enhanced by incorporating reconstruction of contents from the auditory domain. Although it could be a long way to achieve these advanced reconstruction approaches, we present here as a first step in this direction an approach to identification of novel music pieces. In the future, the identification process could be reformulated as a reconstruction using an auditory prior, e.g. large music samples as priors, in analogy with the image prior used in ref.^5^. The use of such priors on different temporal structures provides an alternative way to reconstruct auditory stimulus from brain activity when a direct inverse reconstruction of brain activity to auditory signal is not possible.

**Figure 1:**
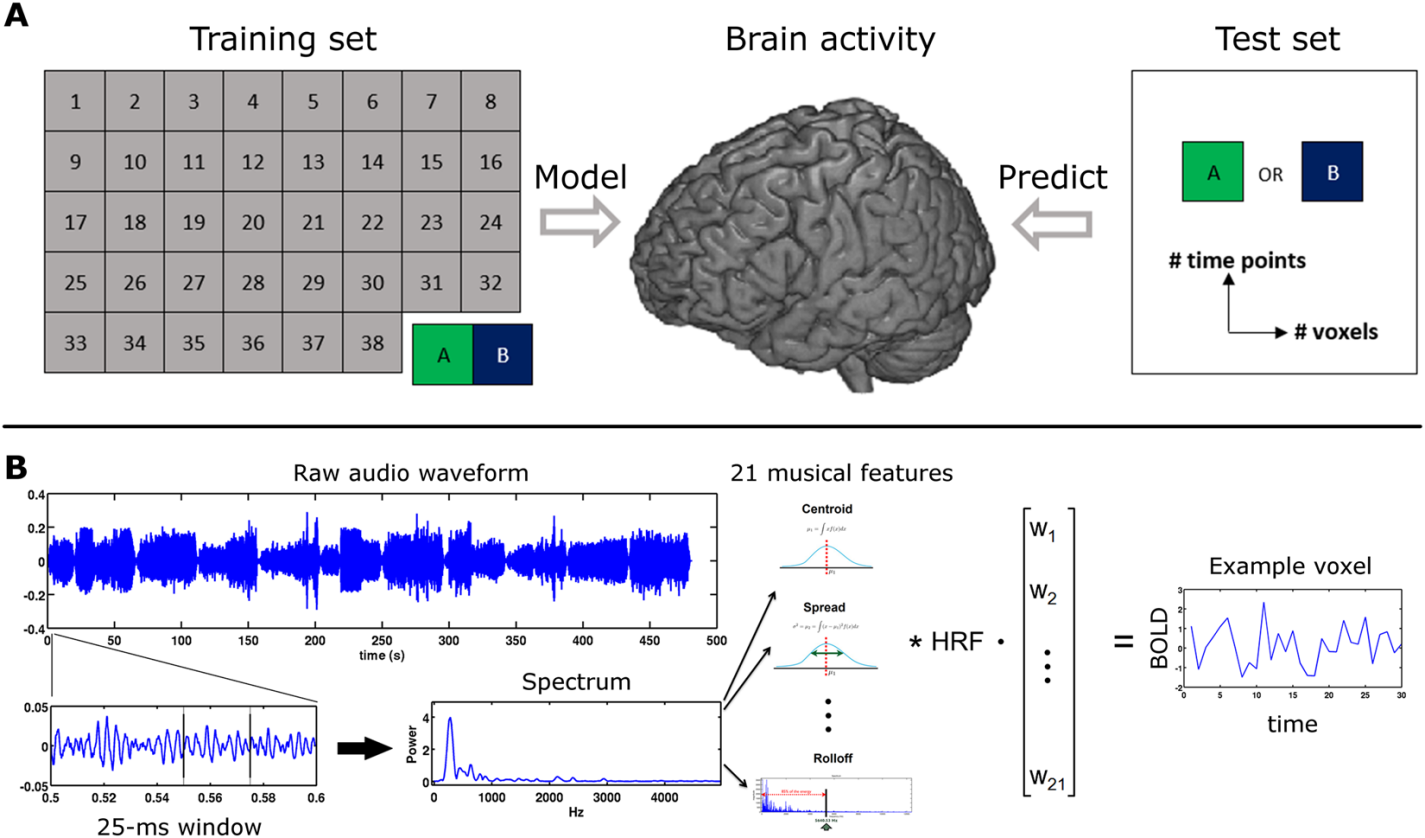
Encoding and decoding model. (**A**) Overall experimental and algorithmic overview: Subjects listened to 40 music pieces (46 s each). In each of the 780 iterations, a voxel-wise encoding model used 38 pieces to estimate 21 musical features weights (training phase). The two left-out pieces were then predicted by the learned model. During the decoding stage, the number of time points and voxels used for identification were iteratively increased. This systematic approach allows a detailed examination of how model performance is related to the temporal and spatial dimensions. (**B**) Voxel-wise encoding model: The raw audio waveforms of the 40 music pieces were used to extract 21 musical features, mostly based on the spectrum of 25-ms windows, such as spectral centroid, spread, etc. (see Methods for details). The musical features were then convolved and resampled to match the fMRI sampling rate (TR = 2 s). Multiple linear regression was used to estimate the weights for each voxel independently (voxel-wise encoding).

Here, we constructed a cross-validation procedure using an established musical feature set comprising low-level as well as higher-level characteristics of musical dimensions like tonality, dynamics, rhythm and timbre for the encoding model and then tested this model by decoding brain activity elicited due to novel musical pieces that were not used to train the encoding model. Six subjects listened to a large stimulus set of 40 different pieces (46 s each) from various genres, yielding a total scanning time of roughly 2 h (four presentations of each piece). The 40 pieces were grouped into four medleys. Next, 21 musical features encompassing rhythmic, timbral and tonal properties (see Methods and ref.^12^) were extracted using the MIRtoolbox^8^ (on different time scales and then were downsampled to the temporal resolution of fMRI data). These features were next used to model the ongoing brain activity in the temporal lobe by multiple linear regression analysis. The coefficients of the encoding model were estimated in a leave-two-pieces-out scheme, with two 46-s music pieces left out. In the decoding stage, we asked which musical features of one of the two pieces that were not used in the encoding model were more likely to predict the measured blood-oxygen-level dependent (BOLD) signal. Overall, 780 pairs (40 × 39/2) were tested. For each pair of music pieces A and B, the algorithm compared if (1) brain activity during piece A was better predicted by using the parameters identified in the encoding model and features of music piece A than by features of the music piece B; and equivalently if (2) brain activity during piece B was better predicted by features of piece B than by features of piece A (see Methods for further details). The overall procedure is illustrated in Fig. 1. Based on this approach, we investigated how distributed information in different areas of the auditory cortices contributes to decoding performance. Thus, we measured and tested explicitly how the coverage (i.e., the number of voxels) relates to decoding accuracy. In addition, we investigated how decoding accuracy varies over time by adding more and more time points from the music pieces to the decoding stage. With this systematic approach, we are able to address how model performance is intrinsically related to the temporal and spatial dimensions. Moreover, with our choice of various musical styles, we can also explicitly test the generalization ability of our model.

## Results

### Significant identification accuracies for all subjects

The combination of encoding and decoding models enabled us to discriminate the temporal and spatial dimensions for each individual and to determine what drives model performance. We therefore varied the number of voxels taken from *a priori* regions, including Heschl’s gyrus and superior and middle temporal gyrus, and determined identification accuracy in the ongoing music piece by adding time points to each musical excerpt. Voxels were added according to the rank of prediction correlations as established during the encoding phase. Identification accuracy was measured as the percentage of correctly identified pieces over the total of 1,560 identifications (see Methods for details).

Identification accuracies of 85% and 84% were obtained for two of six subjects and ranged between 70‒78% for the other four subjects. Overall, model performance was significantly above chance level for all individuals: 76.8% ± 6.5% (mean ± SD) as compared to 62.5% (p < 0.05) obtained by randomly permuting piece labels during the decoding stage (see Methods).

**Figure 2:**
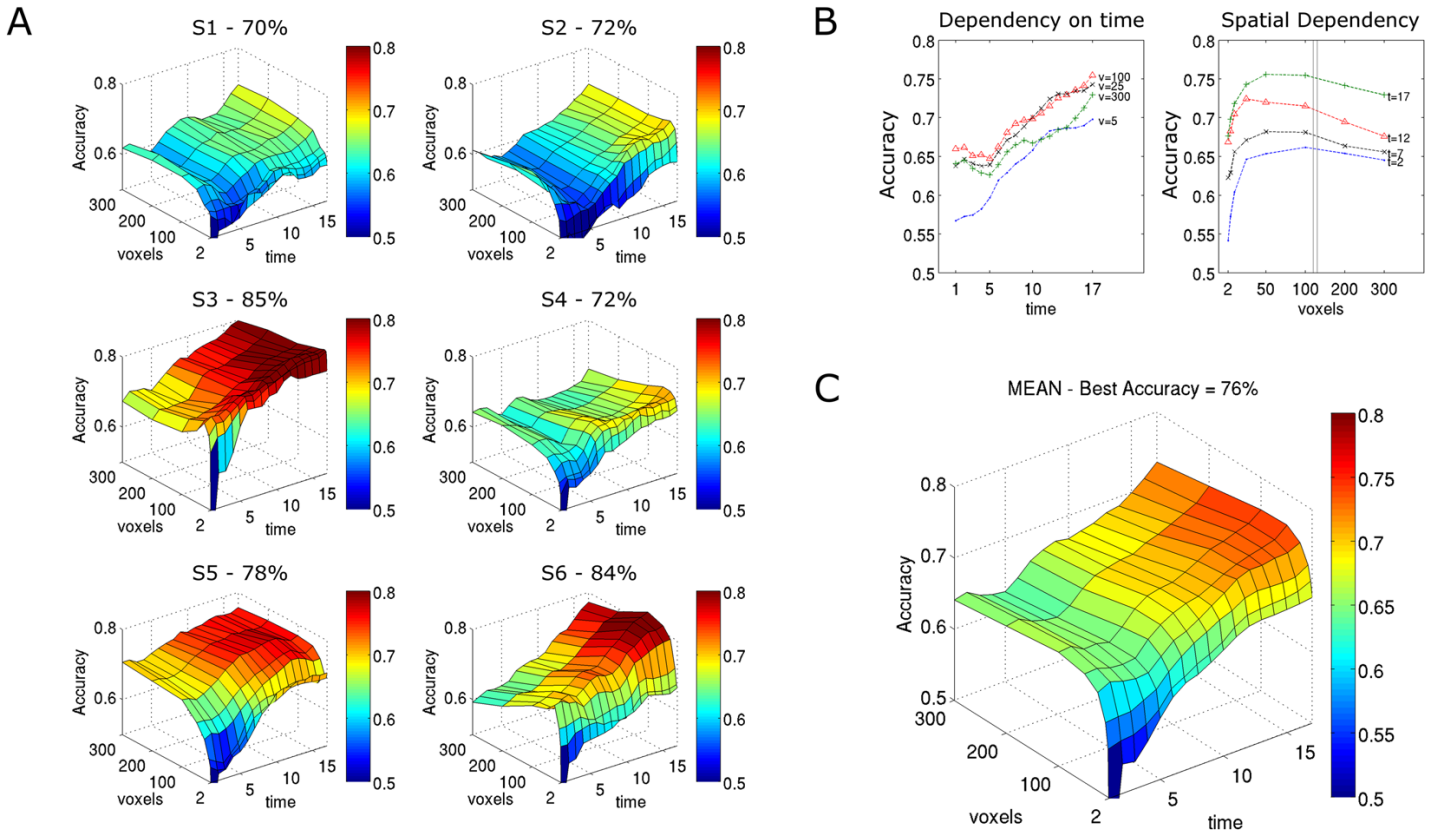
Model performance profiles. (**A**) Identification accuracy for all six subjects (S1–S6) as a function of space (number of voxels) and time. Note, that time ranges from 1–17 volumes as the first and last three volumes of the transition between pieces were left out (TR = 2 s). Highest accuracies were obtained for S3 (85%) and S6 (84%). Model performance profiles show great similarity across subjects, with a steep initial increase in the spatial dimension and a linear increase in the temporal dimension. (**B**) Different role of temporal (left side) and spatial (right side) dimensions (mean values for the six study subjects): model performance saturates at roughly 100 voxels for the spatial dimension after a steep initial increase, whereas the temporal dimension shows a linear relationship. It should be noted that voxel size is arbitrary and thus different voxel sizes may result in different slopes of identification accuracy. (**C**) Mean values of general profile characteristics for the six study subjects.

The temporal-spatial profiles of model performance (i.e., identification accuracy) showed great similarity across subjects (Fig. 2A: individual profiles, Fig. 2B,C: mean profile). Model performance started at chance level (50%) for 2–5 voxels and the first time point and sharply increased in the spatial dimension. Figure 2B further highlights that model performance saturated at approximately 100 voxels for the spatial dimension after the initial increase, whereas the temporal dimension showed a nearly linear relationship for the whole length (46 s) of musical pieces.

### Anatomical distribution of most contributing voxels

**Figure 3:**
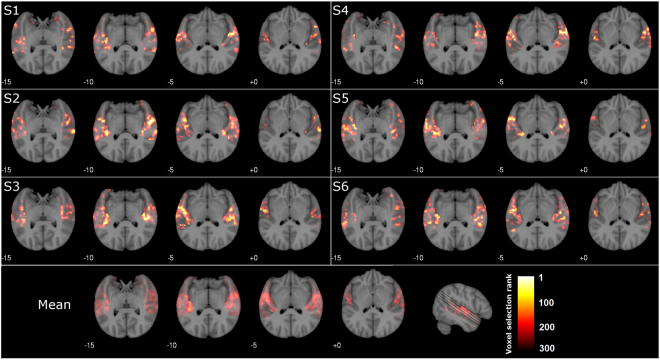
Decoding voxel maps. The voxels used during the decoding stage are shown for all six subjects (S1–S6) on four axial slices displayed with an angle of 34°^37^. The colour indicates the rank during model estimation (highest rank: yellow/white; lowest rank: dark red). Despite individual differences, highest ranked voxels concentrate around Heschl’s gyrus and planum temporale/polare for all subjects (bottom part). The maps indicate that the musical features reflect both lower-level and higher-level auditory processing, as supposedly represented in Heschl’s gyrus and anterior and posterior regions like planum polare and planum temporale, respectively.

The 300 most frequently used voxels for identification are shown in Fig. 3. In each iteration, the voxels were selected according to their training prediction correlations. The most frequent used, i.e., the highest ranked voxels concentrate around Heschl’s gyrus (HG), followed by secondary auditory regions planum temporale (PT), planum polare (PP), and anterior and posterior superior temporal gyrus (STG). Other regions such as middle temporal gyrus (MTG), supramarginal gyrus (SMG), and temporal pole (TP) contributed less to the identification stage.

### Representation of musical features across auditory cortices

To demonstrate the functional subdivisions within the auditory cortex, we calculated the correlations between each musical feature and the BOLD time series of each of the 300 best voxels identified during the encoding stage. Then, we applied principal component analysis (PCA) to these 21 separately estimated correlations to reveal the musical feature representation across the auditory cortices. This approach avoids any bias that could be possibly introduced by collinearity between musical features. Results showed that the first principal component was highly consistent across subjects (Pearson correlation between loadings across all pairs of subjects (N = 6*5/2): *r* = 0.95 ± 0.04), whereas the second principal component showed still high similarity (*r* = 0.77 ± 0.20). Thus, group principal components (PCs) were calculated over concatenated voxels from all subjects. The first two components explained 71% (48% + 23%) of the variance. For the interested reader, we included the third and fourth component in the Supplementary Fig. S2 which explain 11% and 6% of the variance, respectively. Because the best decoding performance was obtained for 100 voxels, we also applied PCA for this reduced number of voxels. Results were substantially the same as those for the larger number of voxels (similarity of first two PCs measured by Pearson correlation coefficient within each subject (N = 6): *r* = 0.96 ± 0.05 and *r* = 0.86 ± 0.17 for PC1 and PC2, respectively).

**Figure 4:**
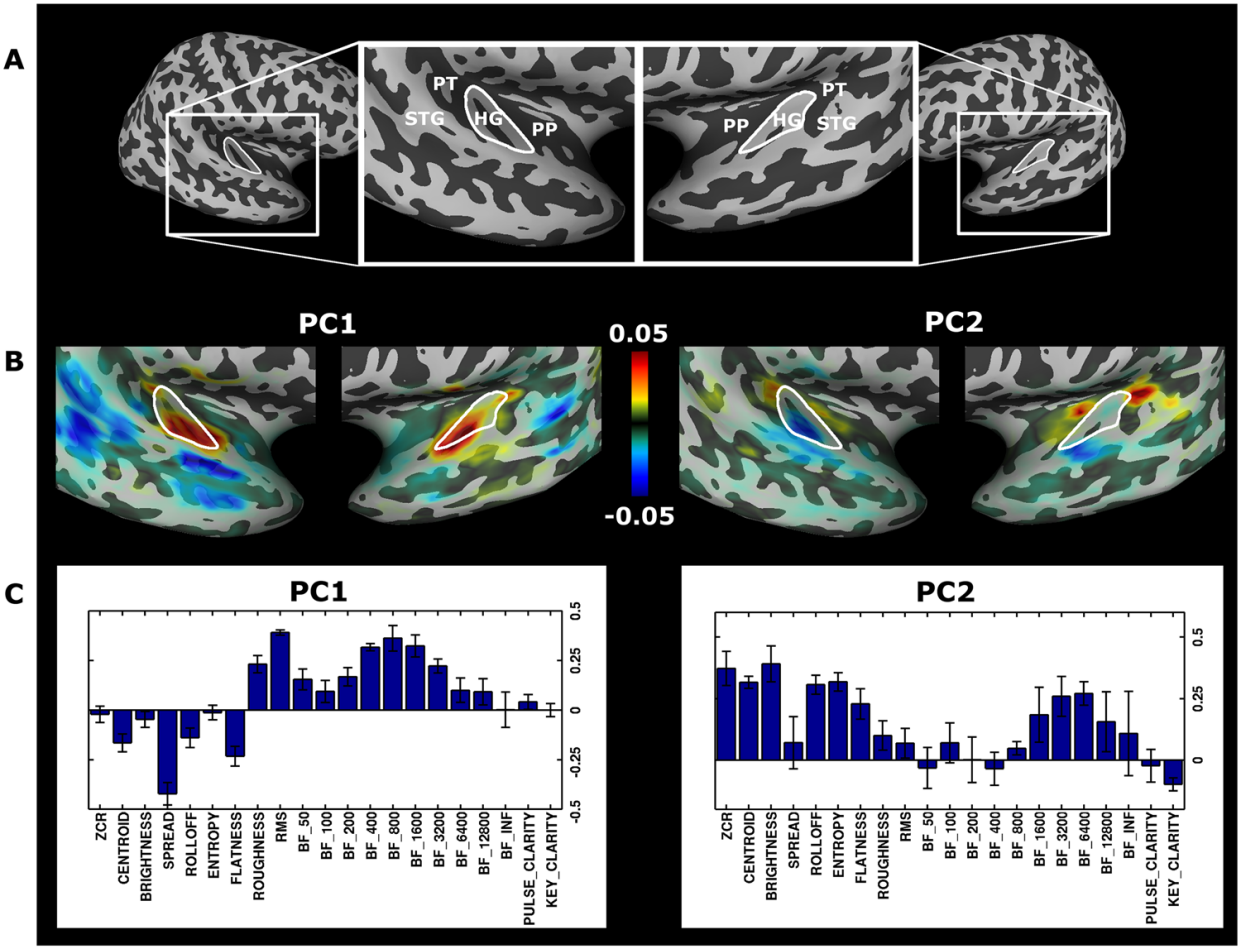
Cortical distribution of musical features. (**A**) Anatomical landmarks on an inflated surface of the temporal cortex of one example subject, showing Heschl’s Gyrus (HG), planum polare (PP), planum temporale (PT) and superior temporal gyrus (STG). (**B**) Overlays show the mean scores of the first two principal components on the right and left hemisphere (HG outline in white). The scores were scaled with the encoding training correlations to emphasize music-responsive voxels. Left: The first principal component (PC1) concentrates positive scores within Heschl’s gyrus (HG). Right: The second principal component (PC2) is mostly represented anterior and posterior to HG, i.e., planum temporale (PT) and planum polare (PP). (**C**) Loadings of PC1 and PC2 (mean bars and SD error bars over subjects). The musical features for PC1 load positively on roughness, root-mean-square energy (RMS) and sub-band flux frequencies between 200 Hz and 1600 Hz (number in axis-labels indicate upper frequency of sub-band, e.g. BF-400 represents sub-band flux between 200–400 Hz) and negatively on flatness and spread (see Musical Feature Extraction section in Methods for details and ref.^8^ for description of musical features). On the other hand, PC2 shows positive loadings on higher frequencies and brightness. These two principal components show the low-frequency preference of the HG and the high-frequency preference of the surrounding regions PP and PT. Interactive visualizations of individual results are made publicly available on institutodor.github.io/mirviewer/S1.

The cortical distribution of the mean principal component scores and their respective loadings are shown in Fig. 4 (see Supplementary Fig. S1 for individual distributions). Despite the known individual anatomical and functional variability of the auditory region^18^, the results show a clear preference of the first principal component representing lower frequencies within Heschl’s gyrus. Further, the musical feature loadings on this component (i.e. positive loading of roughness—a measure for sensory dissonance, root-mean-square energy—a loudness feature and sub-band flux frequencies between 200 Hz and 1600 Hz and negative loadings of flatness, i.e. a description of the smoothness of the frequency distribution) are similar to a mixture of the components termed “activity” and “timbral complexity” in a previous study^12^. The second principal component loads more on higher frequencies and shows similarity to the “brightness” component. This component is most clearly located in anterior and posterior regions of HG, namely PP and PT. Taken together, the cortical distribution highlights the functional architecture of the core regions, represented by low frequencies centred at HG and higher frequencies in the surrounding areas PP and PT^18^.

### Assessment of model sparsity

**Figure 5:**
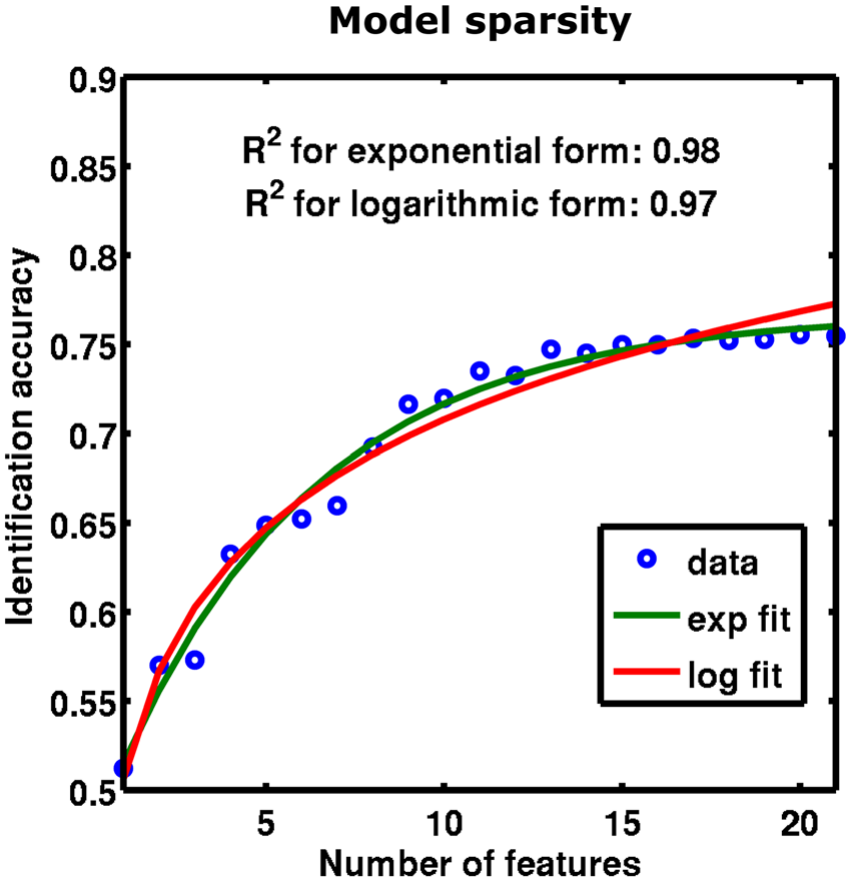
Assessment of model sparsity. Identification accuracy as a function of the number of features used in the encoding model. Accuracy rates are near chance level for one feature, increase sharply afterwards, and remain roughly at the same level after 13 features. Blue dots represent mean values over two orders of increasing features and all subjects (accuracy taken at 100 voxels and full piece length). Green line: fit with exponential function. Red line: fit with logarithmic function.

We tested if a sparser model could reach equivalent decoding performance. More specifically, we assessed how identification accuracy is affected by the number of variables in the encoding model. We applied the identification procedure by varying the number of features from one to 21. Because there is no intrinsic order to the features and to avoid over- or underestimating the effect of the number of variables, we applied the procedure twice in opposite orders of the variance inflation factor. Model performance increased sharply for the first features and only slightly after 13 features (Fig. 5). The relationship between model performance and number of features was slightly better fit by an exponential function than by a logarithmic function.

### Decoding stage robustness: identifying up to 10 pieces

**Figure 6:**
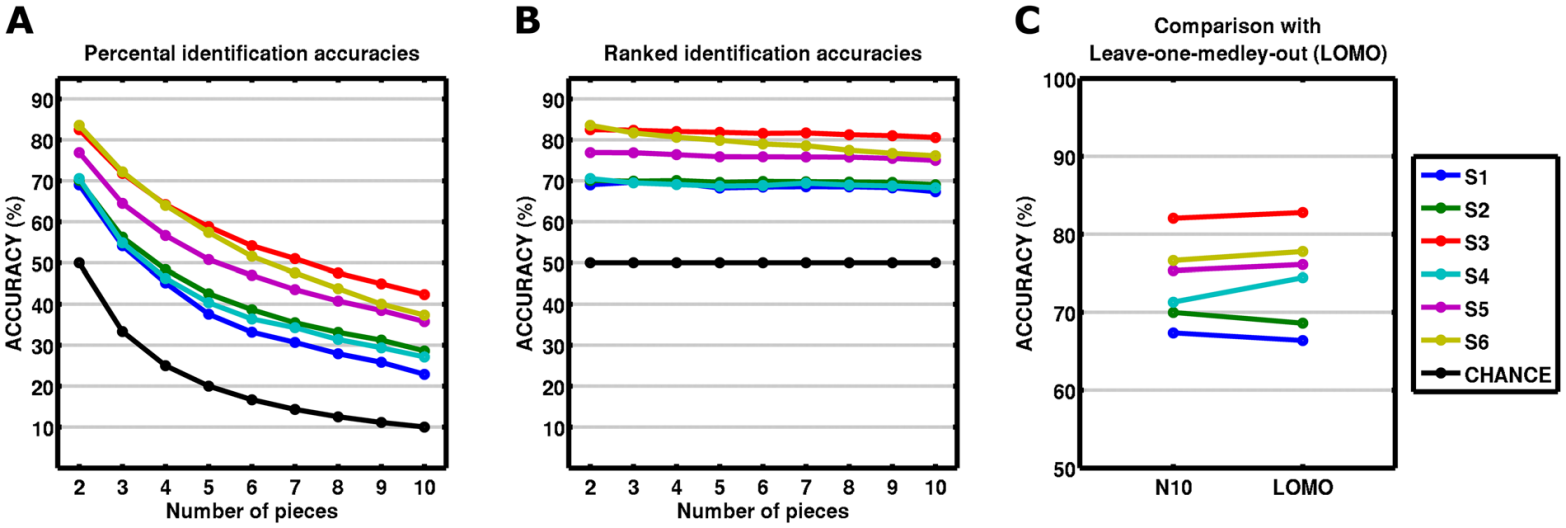
Robustness in identifying one piece out of several pieces (2‒10). Accuracies are well above chance-level for all subjects (S1–S6) when using the original metric for accuracy (**A**) and remain stable when using ranked accuracy (**B**), calculated as (N-rank(predicted))/(N-1). This analysis shows that the decoding approach is not limited to binary identification. (**C**) For the case of N = 10, accuracy was compared for choosing randomly 10 pieces out of all 40 pieces (N10) and for the most stringent leaving-one-medley-out (LOMO) validation scheme, i.e. leaving out a complete functional run. Identification accuracies are at the same level and no systematic difference between these two cross-validation procedures was observed (paired t-test t(5) = −0.86, p = 0.42). This indicates that identification accuracies are not inflated by any temporal correlations that could possibly exist within a functional run.

We asked if our identification procedure is robust to identify more than two pieces at once, i.e., we left out N = 3 to N = 10 pieces out during the encoding stage in order to test these music pieces during the decoding stage. Because the number of possible cross-validation folds increases sharply (N = 3: 9,880; N = 4: 91,390; …), we opted to randomly choose 10,000 iterations for N ≥ 4 due to computational reasons. For the N = 10 case, we additionally left out all 10 pieces of each entire medley at a time, i.e., a complete functional run. The results showed that the decoding stage is not restricted to identifying one piece out of two. Model performance remained well above chance level when using percental identification accuracies and stable for ranked accuracies for identifying one piece out of up to 10 pieces (Fig. 6A,B). Results for identifying 10 pieces of the same medley showed the same level of accuracy as using 10 pieces across different medleys (73.8% ± 5.3% vs. 74.4% ± 6.0%; paired t(5) = −0.86, p = 0.42; Fig. 6C). This result shows that identification is robust also for a completely new fMRI run not used during the encoding stage and thus is not biased by any temporal correlation between training and test sets^19^.

### Shannon entropy

**Figure 7:**
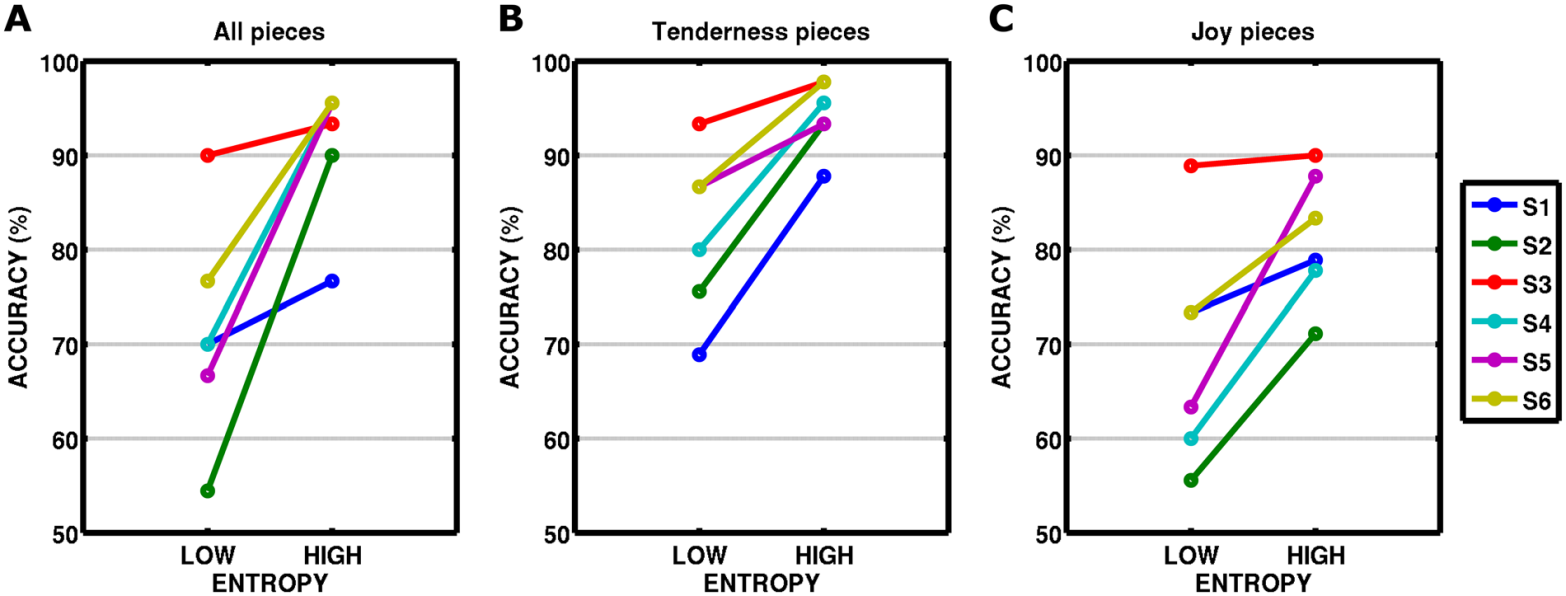
Entropy of pieces explains gain in identification accuracy. (**A**) The whole stimuli set was divided into 10 pieces with low and high entropy. The effect of entropy on identification accuracy is clearly visible. (**B**,**C**) The same effect was observed for the divisions made within the tenderness, and joyful music pieces, indicating that this metric can explain differences in identification accuracy beyond categories. A two-way ANOVA with factors emotional category (tenderness, joy) and entropy (low, high) confirmed main effects for category (F(1,5) = 18.4; p = 0.008) and entropy (F(1,5) = 32.4; p = 0.002).

In a subsequent analysis, we used entropy as a high-level metric to explain additional variance in identification accuracy, based on the hypothesis that identification of a piece might depend on the richness of musical feature changes (i.e., information content) within each piece. For this purpose, Shannon entropy of the feature space was calculated for each piece (see Methods). We hypothesized that the more information, the higher the probability of correct identification. By splitting the test stimuli into two sets of high and low entropy, we could confirm the impact of piece-information-content on model performance, namely entropy as a driving factor of correct identification (Fig. 7A). Identification accuracy among the 45 pairs of the 10 pieces with topmost entropy was 95.5% for three subjects, 93.3%, 90.0% and 76.7% for S3, S2 and S1 (91% ± 7%; mean ± SD across subjects) in comparison to 71% ± 12% (mean ± SD across subjects) for the 10 pieces with least entropy. Because our stimulus set included both tenderness and joyful musical pieces (see Supplementary Fig. S3; and ref.^20^), this result was probed further on separate examination of tenderness and joyful medleys (Fig. 7B,C). A two-way repeated measures ANOVA using permutations on the dependent variable accuracy (aovp function of lmPerm package^21^ in R) with subjects as random factor and within-subject factors emotional category (tenderness, joy) and entropy (low, high) showed main effects for category and entropy (p = 0.0014 for each factor), but no interaction between emotional category and entropy. A post hoc test (pairwisePermutationTest of rcompanion package^22^ in R) showed significant higher accuracies for tenderness than for joyful pieces (p = 0.025; tenderness: 88.1% ± 6.1%; joy: 75.3% ± 8.9%; mean ± SD over subjects). The driving factor of entropy was confirmed by significant higher accuracies for high entropy versus low entropy pieces (p = 0.0009; high entropy: 87.9% ± 4.6%; low entropy: 75.5% ± 9.1%). The higher accuracies for the tenderness pieces can be explained by the higher entropy values in comparison to joyful pieces (paired t(38) = 3.56; p = 0.001).

### Lateralization

As there is evidence for right hemispheric lateralization of certain aspects of music^23,24^, we checked the lateralization of the decoding voxel maps presented in Fig. 3, by comparing the number of best voxels in the left and right hemisphere. The best 100 voxels of the 6 subjects did not indicate lateralization (paired t-test t(5) = −0.28, p = 0.79). Similarly, no lateralization was found when using the best 300 voxels: t(5) = 0.66, p = 0.54).

## Discussion

In this study, we investigated fMRI brain responses to 40 musical pieces of various genres with a two-stage encoding-decoding model that provided a description of the temporal evolution and spatial location of voxels belonging to auditory cortices critical for identifying musical pieces. Our approach was capable of identifying musical pieces with improving accuracy across time and spatial coverage. Specifically, we showed that the distributed information in auditory cortices and the entropy of musical pieces enhanced overall identification accuracy up to 95%. It is worth noting that these results were obtained for a heterogeneous stimulus set (Classical Music, Rock, Pop, Jazz, and Folk with and without lyrics) including distinct emotional categories of joy and tenderness. Our results contribute with novel insights on the spatiotemporal organization of complex auditory representations stored in the human auditory cortex, and open possibilities for future applications, such as decoding subjective auditory contents during inner speech^17,25^ or enabling BCI-based neuromodulation for patients suffering from auditory hallucinations^15^, based on reconstructed auditory activity. This approach could also be used for the reconstruction of imagined music, by incorporating an auditory prior in the decoding model, based on large databases of music samples, in analogy to the image priors employed for natural image reconstruction^5^.

We extended the applicability of encoding and decoding models from the visual and semantic domains^1,4,26^ to the musical domain. Previous studies concentrated either on the encoding models of principal components of musical features^12,13^ or on the direct decoding of these components from brain activity^14^. Other studies focused on categorical differences between music styles and their associated similarities between neural and musical feature representations^27,28^. Similarly, a recent study investigated the multivariate representations of categorical genres by decoding the neural representations from brain responses alone and used encoding models of melodic features in a separate searchlight analysis^29^. Here, we added a second decoding phase to the first encoding stage that provided the ability to not only identify novel musical pieces (i.e., that were not used during the encoding stage), but also explicitly investigate how model performance relates to the temporal and spatial dimensions. To the best of our knowledge, no other study has ever addressed this explicit combination of encoding and decoding models or has investigated model parameters as we have in our study. We found the expected relationship between time and identification accuracy. For the spatial dimension, by adding more voxels during the decoding stage, we were able to accurately determine to which extent the model benefits from a multivariate approach. Interestingly, after a sharp increase in identification accuracy for the first 50‒100 voxels (with the original voxel size of 3.75 × 3.75 × 4.75 = 66 mm³), model performance saturates and declines as the number of voxels increase. Importantly, this inflection point of model performance, i.e., where performance stops to increase and starts to decline, can be used to make statements about practical significance as opposed to statistical significance testing. Instead of an arbitrary choice of a p-value threshold^30^, we argue that only voxels contributing positively to model performance should be considered practically significant. Thus, it is the combination of encoding/decoding models with the systematic investigation of model performance that goes beyond statistical significance testing and provides insights into practical significance.

The absolute identification accuracies found in our study are comparable to or even higher than those reported in other studies in the auditory^7,31^ and musical^14,28^ domains. A recent publication also presented robust decoding accuracies beyond the chance-level^29^. However, the decoding analysis measured brain response similarity across repeated stimulus presentations without building on an encoding model. In addition to this central methodological difference, the direct comparison of absolute accuracies is not straightforward as fMRI data were acquired on an ultra-high-field scanner (7T) and the specific cross-validation procedure was different.

Our main findings are based on a binary classification procedure and a within-subject cross-validation procedure. A recent article^19^ warned that this procedure could generate biased accuracies, especially when individual observations (i.e., data of volumes from the same fMRI run) are left out. However, our approach leaves out a block of data (entire musical piece of 46 s) instead of individual observations (volumes) and remains robust after leaving up to 10 pieces out for the validation set. Importantly, identification accuracies also remain at the same level when leaving out a complete fMRI run (10 pieces of the same fMRI run, Supplementary Fig. S2). This robustness indicates that identification accuracies in our study are not inflated by our within-subject cross-validation procedure, probably due to the leave-one-block(piece)-out validation and the special care taken to not include the transition between the musical pieces during the decoding stage (see Section Decoding Stage in Methods).

Our investigation of model sparsity confirmed the importance of using rich stimulus descriptions and multiple variables, here in the form of musical features. Although identification accuracy increased monotonically with the number of features, the dependence of model performance on the number of features could be better modelled with an exponential function than a logarithmic function for our specific musical feature set. At first, this might indicate that further increasing the number of features would not improve identification accuracy. However, we used a well-established and homogeneous feature set comprising lower-level as well as higher-level characteristics of musical dimensions. Future studies should investigate if other musical feature types could enhance model performance. Candidates for these additional features could be found in the large musical information retrieval field^10,32,33^. However, the choices are not straightforward and include options like window length, descriptive statistics of neighbouring windows for temporal integration, and ultimately, more sophisticated analyses of musical structure based on similarity measures.

Our study shows some similarities with the cross-validation approach used by ref.^14^ that classified longer segments of music. However, in their study, subjects only listened to one musical style (B-side of the album Abbey Road by The Beatles) and the musical feature components were used as the dependent variable. In contrast, our stimuli stem from a broad range of musical styles and genres (Classical Music, Rock, Pop, Jazz, and Folk with and without lyrics) and included samples from two different emotional categories that differ considerably in their musical features. Although it has been shown that different genre categories induce distinguishable brain patterns^29^, our approach was able to generalize across genres and styles. This indicates that our model captures implicitly the genre differences, which is important in terms of predicting pieces from novel genres.

The detailed investigation of model performance and musical piece entropy was motivated by the observation that the musical pieces contained different amounts of variation. It is intuitively clear that the more variation in musical features there is within a musical piece, the higher the chance to successfully predict the piece. Technically, the prediction is measured by the correlation between predicted and measured responses for each piece. Thus, concerning the temporal dimension, the prediction depends necessarily on the variation beyond the mean of a piece. In the extreme case of zero variation in the stimulus space, the temporal prediction would be constant and only the spatial dimension could contribute to the identification, i.e., only differences in encoding weights across voxels would contribute to spatial variation. The results of our fMRI study suggest that the tenderness pieces had favourable characteristics for accurate predictions, probably due to the preservation of slow temporal variations in the convolved and downsampled musical features inside our model. In sum, the temporal information content of the musical features, as measured by entropy, seems to be a driving factor for enhancing identification accuracy.

Finally, the musical features used in our encoding model allowed us to determine their underlying internal representation and anatomical distribution. We found that not only the Heschl’s gyrus, but also more anterior and posterior regions are well predicted with our musical feature set. Similarly, ref.^34^ showed that maximal brain responses to musical stimuli were located in anterior and posterior regions and that musical responses were less selective when compared to speech, indicating less functional specialization for music. Thus, it is not surprising that a large, yet limited number of voxels contributed to model prediction, including Heschl’s gyrus, planum temporale, planum polare, and superior temporal gyrus. These regions are all related to musical responses and their role has been addressed in several studies^35–39^. Moreover, principal component analysis of the musical feature correlations revealed that the first principal component explained much of the variance across voxels in Heschl’s gyrus and anterior and posterior superior temporal gyrus. Importantly, the first PC shows high loadings for lower to middle frequencies (200–1600 Hz), sound energy/loudness (RMS), roughness (a measure for sensory dissonance) and negative loadings for spread (dispersion of the frequency spectrum), flatness (smoothness of the frequency distribution) and centroid, therefore, capturing aspects of already perceptual validated components, namely activity and timbral complexity^12^. Activity can be interpreted to be represented by the bipolar terms ‘strong-weak,’ ‘soft-hard’, and ‘high energy-low energy’^40^, whereas timbral complexity was validated perceptually directly by a rating scale with ‘low/high timbral complexity’^12^. Complementary to this, the second principal component might represent the concept of brightness related to the higher-level perceptual dimensions ‘colorful-colorless’, ‘bright-dark’^40^). In addition to the higher-level concept, this component shows also high loadings on higher frequencies of the lower-level auditory sub-band flux features and is predominantly located at the planum temporale and planum polare, consistent with the representation of high frequencies in this region as demonstrated in numerous tonotopy studies^18,41–48^. These studies generally employed pure tones at different frequencies ranging from 200–8,000 Hz to precisely locate primary tonotopic gradients, that were found to be largely consistent across laboratories and acquisition/analysis types (for a review on this topic, see^18^). In addition, RMS (sound energy) loaded more on PC1 and had higher scores on HG, whereas other features like brightness, zero-crossing-rate (ZCR) and centroid of PC2 scored higher at PT e PP, suggesting that these sub-regions might have more functional relevance for these acoustic features. These findings and interpretations are still preliminary and need confirmatory evidence from future studies, which should also investigate further musical feature combinations that might reveal additionally functional dimensions and divisions in auditory cortices.

Our model showed no hemispheric preference for musical processing. This lack of lateralization for music features is compatible with the findings of ref.^34^, who only found slight lateralization for specific components representing fine-grained temporal and spectral changes, but not for the musical component. This is consistent with ref.^23^, who related hemispheric differences between fine spectral and temporal resolution to right-hemispheric music preference. Therefore, our finding is not surprising as our model did not use any features specialized for fine spectral or temporal changes that potentially could highlight hemispheric differences. Still in the context of the employed musical features, one could ask which other auditory processes could also be represented by our model. Although previous studies related the musical features used in our model to higher order percepts such as timbral complexity^12^, this does not exclude the possibility that many of the low-level features, especially the sub-band flux features would also describe other acoustic stimuli, such as ripple noise stimuli. Also, the findings of lower frequency representation in HG (PC1) with surrounding higher frequency (PC2) anterior and posterior likely reflect lower-level auditory processes. However, other features with high loads in PC2 also indicates the presence of a timbral dimension. Future studies should extend these findings by employing musical stimuli and feature sets that reflect different acoustic, timbre, tonal and perceptual dimensions and investigate further the relationship between these important aspects.

Brain models applied to fMRI data are limited by both noise in the measurements and the relatively coarse spatial resolution. However, recent advances in MRI technology allow higher spatial resolutions that can even address representations across cortical columns. These recent developments are especially interesting as they also request for more sophisticated musical feature sets that can account for the finer representations in these higher resolved spatial dimensions. Thus, there is certainly large room for improving spatial and temporal accuracy and models that can capture more precisely the rich information contained in natural musical stimuli, including higher-level perceptual, semantic and emotional features. Taken together, our findings contribute to the understanding of complex auditory representations and highlight the importance of combined multivariate encoding/decoding approaches for optimizing model performance. On this basis, future developments and applications can be built that ultimately could enable the treatment of patients with neuropsychiatric disorders such as auditory hallucinations.

## Methods

### Participants

Six healthy volunteers participated (age: 30.8 ± 7.8 years [mean ± SD]; five women and with musical experience, i.e., playing an instrument or singing–age of onset of musical training 6.7 ± 2.1 years; years of formal music training 12.8 ± 7.7 years, self-report of practice hours on their instruments/voice across lifetime summed over all instruments [years × hours per week × 52: 4,424 ± 4,198). All participants had normal hearing and no history of psychiatric or neurological disorders. Written informed consent was obtained from all participants. The study was approved by the Ethics and Scientific committees of the Copa D’Or Hospital, Rio de Janeiro, Brazil (No 442.648) and all experiments were performed in accordance with relevant guidelines and regulations.

### Stimuli

Forty pieces with duration of 46 s from different genres (Classical Music, Rock, Pop, Jazz, and Folk with and without lyrics, see Supplementary Table 1) were selected for the fMRI experiment. The selection of these emotional categories was motivated by the fact that both emotions are positively valenced and represent two key dimensions of musical emotion while having a sufficient number of examples for each dimension. We refrained from including more dimensions to keep the duration of experiments within a comfortable range for participants. The emotional categories were confirmed for each piece by four participants during the selection process prior to the experiment and rated by all participants during the experiment (see Supplementary Fig. S3). All pieces were normalized to have the same volume (RMS normalization of Adobe Audition). Next, the 40 pieces were arranged into four different medleys concatenated with a linear fade-out and fade-in of 1 s each and no further rest period. In addition, to provide a more ecological setting, we separated the medleys into two joy- and two tenderness-evoking medleys, the order of music pieces was fixed (see Supplementary Table 1).

### Experimental protocol

Each subject listened to the four medleys prior to the experiment and was asked to perform either one of the following two tasks: subjects should feel the emotion evoked by the piece (1) or perform an analytic task, i.e., tracking of harmony changes that prevented them from feeling the emotion (2). Training these tasks also made the music familiar to the participants prior to the scanning sessions. FMRI data were collected from each subject during four sessions on different days. During each session, subjects listened to the four 8-min medleys (in 4 runs), including a 20-s “warm-up” piece (total scanning time during music listening: 2 h 8 min per subject). Before each medley, subjects were instructed to perform either the analytical or the feeling task so that they performed twice each task on each medley during the four scanning sessions/days. The presentation order of different medleys was counterbalanced in different sessions for each participant. The analysis aggregated the data over the two tasks; the effect of the task is beyond the scope of this work. The musical stimuli were delivered with MRI-compatible headphones (MR-Confon, www.mr-confon.de) after equalizing the unequal frequency representation of the headphones with the equalizer function provided in Audacity (http://www.audacity.de). Prior to the experiment, the volume level was adjusted individually to provide the best subjective experience using a 20-s fMRI sequence together with normalized stimuli.

### Sequence parameters

Functional images were acquired with a 3T Achieva scanner (Philips Medical Systems) using a T2*-weighted echoplanar (BOLD contrast) sequence (TR = 2000 ms, TE = 22 ms, matrix = 64 × 64, FOV = 240 mm, flip angle = 90°, voxel size 3.75 mm × 3.75 mm, slice thickness = 3.75 mm, gap = 1 mm, 24 slices; 245 volumes per run, four runs on a scanning day). Slices were sampled in equidistant mode to provide optimal musical experience (the low number of slices and the equidistant mode produced a constant background noise that resulted in an overall lower perceived scanning noise). The first 10 volumes corresponded to the warmup piece and the last five volumes were added to account for delays in the hemodynamic response function. Total functional scanning time per session was 32 min. A SENSE factor of 2 and dynamic stabilization were used.

Simultaneous to the fMRI sequence, we measured respiration, electrocardiogram (logged by the Philips Achieva scanner), and galvanic skin response (Brain Products, Germany).

### Data preprocessing

All fMRI volumes of the four sessions of each subject were realigned to the first volume of the first run of the first session using the FMRIB Linear Image Registration Tool (FLIRT) from FSL^49^. Low-frequency drifts were removed with a Savitzky-Golay filter of 242 s and polynomial order three^4^. Realignment was inspected visually to confirm successful motion correction. Additionally, we used retrospective image correction^50^ to remove physiological noise in the BOLD signal. The phases of respiration and heartbeat cycles were determined and modelled with a fourth and third-order Fourier expansion, respectively, together with a first-order interaction, yielding 18 parameters. Also, we modelled the respiration and cardiac response function as suggested by previous studies^51,52^. The corresponding confound regressors were created using the Matlab PhysIO Toolbox^53^ with open source code available as part of the TAPAS software collection: http://www.translationalneuromodeling.org/tapas/). Galvanic skin response was artifact-corrected in BrainVision Analyzer (Brain Products) and convolved with the standard hemodynamic response function of SPM12. In total, 21 physiological confound regressors were subtracted from the BOLD signal after estimating their contribution by multiple linear regression analysis. The next two steps of preprocessing included global signal correction and temporal Gaussian filter with a width of 5 s^12,54^. Finally, each run was normalized to have zero mean and unit variance before entering the encoding analysis.

### Musical feature extraction

We used an approach similar to that of ref.^12^ for acoustic feature extraction. Twenty-one acoustic features capturing timbral, rhythmical, and tonal properties were extracted from the four medleys using the MIRtoolbox^8^. The features were extracted using a frame-by-frame analysis approach commonly used in the field of Music Information Retrieval (MIR). The duration of the frame was 25 ms with a 50% overlap between two adjacent frames for the timbral features and 3 s with a 67% overlap for the rhythmical (pulse clarity) and tonal (key clarity) features. Timbral features included zero-crossing-rate, spectral centroid, brightness (high energy–low energy ratio divided at 1,500 Hz), spectral spread, spectral rolloff, spectral entropy, spectral flatness (Wiener entropy), roughness, root-mean-square (RMS) energy, and Sub-Band Flux (10 coefficients for 10 frequency bands [0–50 Hz; 50–100 Hz; 100–200 Hz; 200–400 Hz; 400–800 Hz; 800–1,600 Hz; 1,600–3,200 Hz; 3,200–6,400 Hz; 6,400–12,800 Hz; > 12,800 Hz]). Features were convolved with the double-gamma hemodynamic response function with parameters for the auditory response as described in ref.^55^. After convolution features were downsampled by retaining one sample for every 160 samples to match the sampling rate of the fMRI data (TR = 2 s). The same Savitzky-Golay filter applied to fMRI data was used for the convolved musical features. All four medleys were normalized as a whole rather than individually to have zero mean and unit variance.

### *A priori* mask for voxel selection

An *a priori* mask was built from the AAL atlas including regions 79‒86, i.e., Heschl’s gyrus, superior and middle temporal gyrus, and superior temporal pole. The supramarginal gyrus was included partly, as the mask was dilated with “fslmaths -dilM 7” to be permissive for the voxel selection process, yielding 4,654 voxels at the functional resolution of 3.75 × 3.75 × 4.75 mm³. Next, the mask was transformed to the individual space, using the inverse transformation estimated from the segmentation of the anatomical image (as implemented in SPM12), which had previously been co-registered with the functional images. The mask was used in the main analysis as described in the following section.

### Encoding analysis

The voxel-wise encoding model of the musical features was estimated on the training set by multiple linear regression analysis with ordinary least squares method. Regularization was not needed because the number of data points (4 scanning sessions × 38 pieces in training set × 23 TRs for each piece (46 seconds) = 3,496 volumes) was orders higher than the number of model parameters (21). In the training phase, volumes of all runs and sessions were aggregated, except the volumes of the two pieces that were left out for the test phase of the leave-two-music-pieces-out cross-validation scheme. Please note, that data of the repeated presentation of the same music piece were not used in the training data.

### Voxel selection

To establish the voxel selection order for the decoding stage, inner training predictions were determined with a five-fold cross-validation scheme based on the training set that consisted of 3,496 data points (leaving-out two pieces). Therefore, the feature selection was based entirely on the training data, independently from the test data (and thus avoiding circular statistics). For each voxel, weights were estimated using 80% of the training set (2,977 time points) and then used to predict the BOLD time series of the 20% left-out data points (699 time points). Temporal correlations between the measured BOLD signal and the predicted time series were then averaged across the five folds. Voxels were ranked according to this calculated average of the five prediction correlation folds and used in this order during the decoding stage.

### Decoding stage (Identification)

To eliminate any boundary effect, the volumes of the transition between pieces were left out (three volumes corresponding to the beginning and three volumes corresponding to the end of the piece, counted after considering three volumes for the hemodynamic delay). During the identification stage, the test pieces were predicted individually, yielding a total of 1,560 predictions for all two of 40 pairs (780 pairs, two test pieces). The BOLD signal of the four presentations of the same piece was averaged prior to prediction, yielding a single representation of each piece to predict. Predictions were evaluated using Pearson correlation coefficient by concatenating the spatial-temporal dimensions to a one-dimensional vector. A piece was considered to be correctly identified if the prediction correlation was higher with the correct musical features than with the non-correspondent features of the other piece in the test set.

The null distribution of identification accuracy was obtained by permutation tests. The same decoding procedure as described above was applied, however, during the identification the labels of the pieces were randomly permuted. For 100 voxels and complete stimulus length, the p-value of 0.05 corresponded to 62.5% identification accuracy.

### Principal component analysis: Representation of musical features across auditory cortices

The representation of musical features across voxels was assessed with a principal component analysis. To avoid any bias possible introduced by any collinearity between the musical features, temporal correlations were calculated separately between each musical feature and the BOLD time series of each of the 300 best voxels identified during the encoding stage. Then, we calculated the principal components using the function princomp (MATLAB®) over these 21 separately estimated correlations to reveal the musical feature representation across the auditory cortices. The input matrix for the principal component analysis was of size [300 × 21] for each subject. The score matrix returned by the princomp function contains the scores for each voxel for the corresponding principal components represented in the columns. The first and second column were used for visualizing the scores of the first and second principal component, respectively, as shown in Fig. 4B. The first two columns of the loadings (COEFF matrix) of the musical features on the first two principal components are shown in Fig. 4C. We concentrate in the main text on the first two principal components which explain together about 71% of the variance across the 300 voxel weights. However, as parallel analysis and Velicer’s MAP test^56^ indicated four important components, we included the third and fourth component in the Supplementary Fig. S2. The same analysis was repeated for only the best 100 voxels to assess the similarity of the principal components between the larger (300 voxels) and the smaller (100 voxels) input space (within subject). Similarity across individual PCs was calculated by Pearson correlations between loadings of all pairs of subjects. To further confirm the correspondence between the principal components across subjects, group principal components (PCs) were calculated over concatenated voxels from all subjects yielding an input matrix of size [1800 × 21]. The Gale–Shapley stable marriage algorithm confirmed the same order of the components by matching each of individual components to the group PCs.

### Entropy calculation

Shannon entropy of the feature space was calculated for each piece *X*. First, the similarity between musical feature vectors at all possible time point pairs over the entire stimulus set was calculated by Pearson correlation coefficient. Next, the range of these similarity values was divided into 10 equally spaced bins. These bins were then used to determine Shannon entropy^57^ for each piece, using all time point pairs belonging to this piece:

1 $$H(X)=-\sum _{i=1}^{10}{p}_{i}lo{g}_{2}{p}_{i}$$

where *p*_*i*_ is the frequency of a similarity value between time points of piece *X* in bin *i*.

In sum, this metric reflects the variability of similarity values between musical features at all time point pairs within a piece.

### Voxel dimensions for analysis and visualization

All imaging analyses were performed with the original voxel dimensions in the subject’s space. For display purposes, results were resampled to 1 mm³ and transformed to MNI standard space using the new segmentation algorithm of SPM12 (rev 6470). The visualization of inflated surfaces was done with pycortex (http://github.com/gallantlab/pycortex)^58^ using trilinear interpolation of the original voxel dimensions. Functional to anatomical alignments were checked visually for each subject by overlaying Heschl’s gyrus (extracted from the Harvard-Oxford-Atlas), transformed onto the functional reference volume of each individual.

### Data availability

The datasets generated and analysed during the current study are available from the corresponding author on reasonable request. Dynamic visualizations of individual results are available at institutodor.github.io/mirviewer/S1.

## Electronic supplementary material

Supplementary Information
