# Supplementary material for: Identifying musical pieces from fMRI data using encoding and decoding models
Source: Sci Rep. 2018 Feb 2;8:2266. doi: 10.1038/s41598-018-20732-3 (PMC5797093; doi:10.1038/s41598-018-20732-3)
Supplement: Supplementary file 1 — Supplementary Information [file 41598_2018_20732_MOESM1_ESM.pdf]

# SUPPLEMENTARY MATERIAL

**Title:**

Identifying musical pieces from fMRI data using encoding and decoding models

**Authors:**

Sebastian Hoefle,[1,2] Annerose Engel,[1,3,4] Rodrigo Basilio,[1] Vinoo Alluri,[5,6] Petri Toiviainen,[5] Maurício Cagy,[2] Jorge Moll,[1,*]

**Affiliations:**

[1] Cognitive and Behavioral Neuroscience Unit and Neuroinformatics Workgroup, D'Or Institute for Research and Education (IDOR), Rio de Janeiro, Brazil
[2] Biomedical Engineering Program, COPPE, Federal University of Rio de Janeiro, Rio de Janeiro, Brazil
[3] Day Clinic for Cognitive Neurology, University Hospital Leipzig, Germany
[4] Max Planck Institute for Human Cognitive and Brain Sciences, Leipzig, Germany
[5] Finnish Centre for Interdisciplinary Music Research, Department of Music, Art and Culture Studies, University of Jyväskylä, Finland
[6] International Institute of Information Technology, Gachibowli, Hyderbad, India

**Corresponding Author and Lead Contact:**

Jorge Moll
D'Or Institute for Research and Education (IDOR)
Rua Diniz Cordeiro, 30
22281-100
Rio de Janeiro, Brazil
Tel +55 21 3883-6000
e-mail: jorge.moll@idor.org

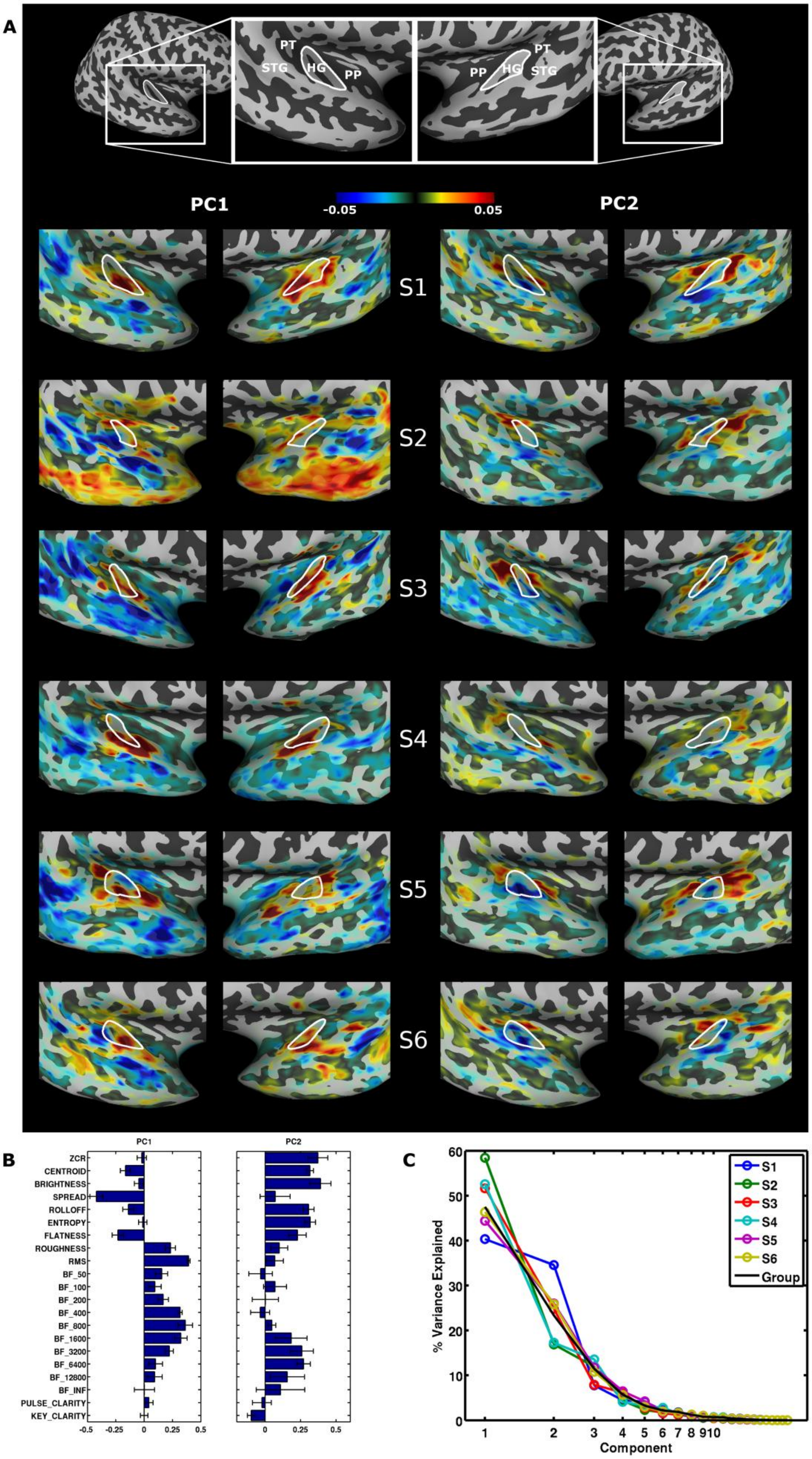
A
PT
STG
HG
PP
PP
HG
PT
STG
PC1
-0.05
0.05
PC2
S1
S2
S3
S4
S5
S6
B
PC1
PC2
ZCR
CENTROID
BRIGHTNESS
SPREAD
ROLLOFF
ENTROPY
FLATNESS
ROUGHNESS
RMS
BF_50
BF_100
BF_200
BF_400
BF_800
BF_1600
BF_3200
BF_6400
BF_12800
BF_INF
PULSE_CLARITY
KEY_CLARITY
-0.5 -0.25 0 0.25 0.5
0 0.25 0.5
C
S1
S2
S3
S4
S5
S6
Group
% Variance Explained
0 10 20 30 40 50 60
1 2 3 4 5 6 7 8 9 10
Component

**Supplementary Figure S1. Individual cortical distributions of musical features.** Related to Fig. 4.

(A) Spatial representations for all six individuals S1 to S6 of the first and second principal component (PC1: left, PC2, right) of the musical feature space. Overlays show the principal component scores on the individual inflated surface centered at Heschl's gyrus (white outline). Scores were scaled with correlations obtained from the encoding stage to highlight music-responsive voxels.

(B) Loadings of first (PC1) and second (PC2) principal component. Bars represent mean loadings over subjects and error bars represent standard deviation across subjects. The components showed high similarity across subjects (Pearson correlation between loadings across all pairs of subjects (N=6*5/2): $r$ = 0.95±0.04 for PC1 and $r$=0.77±0.20 for PC2).

(C) Variance explained by the components. The first two components explained 71% (48% + 23%) of variance across voxels.

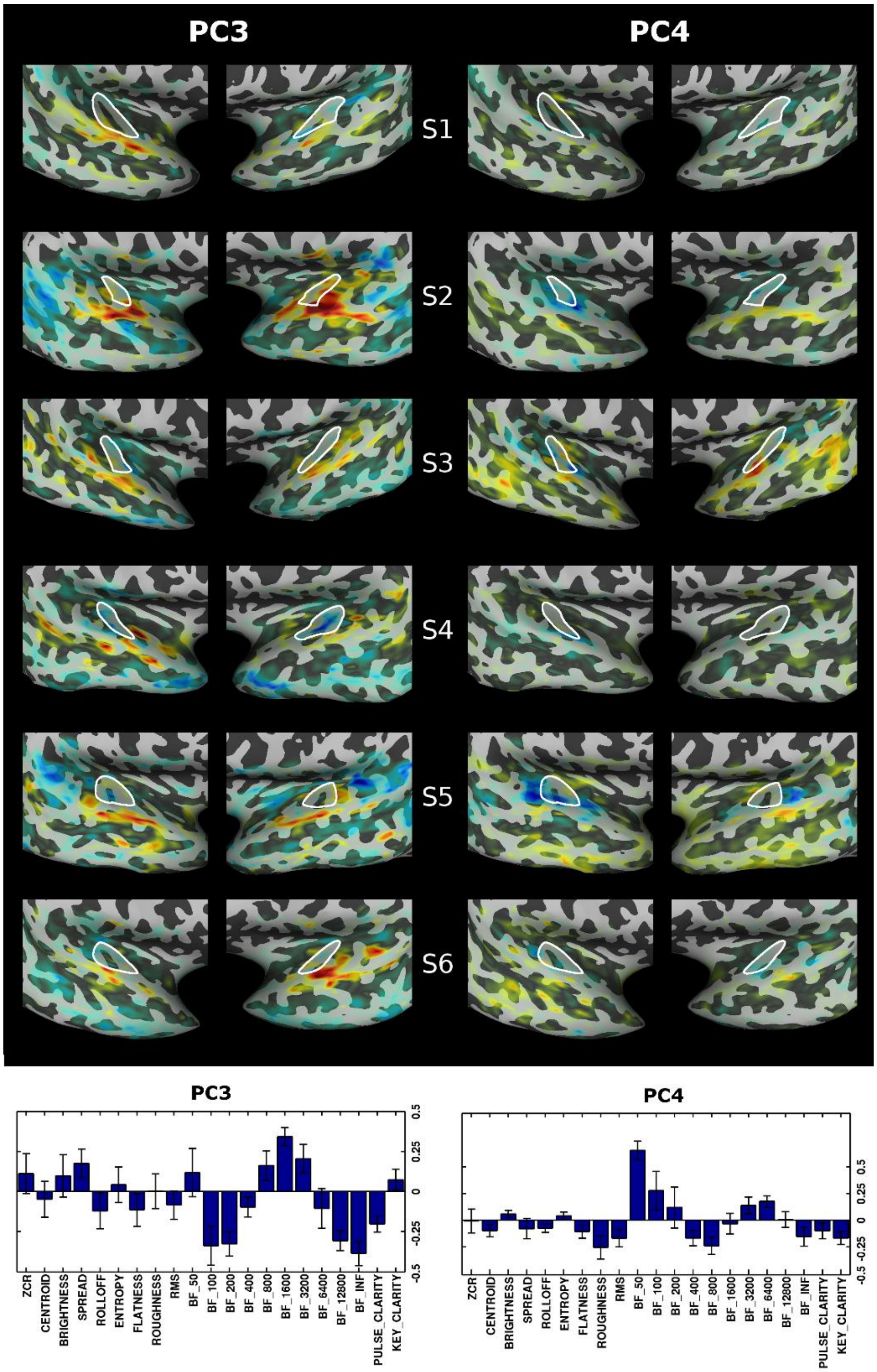


**Supplementary Figure S2. Individual cortical distributions of musical features for 3rd and 4th principal components.** Related to Fig. 4

The third component PC3 is located at the crown of the superior temporal gyrus for most of the subjects. Due to its location, it could represent both speech and musical contents (see Component 5 and 6 in ref. 1). PC4 shows more variation across individuals, such that it is difficult to interpret its meaning.

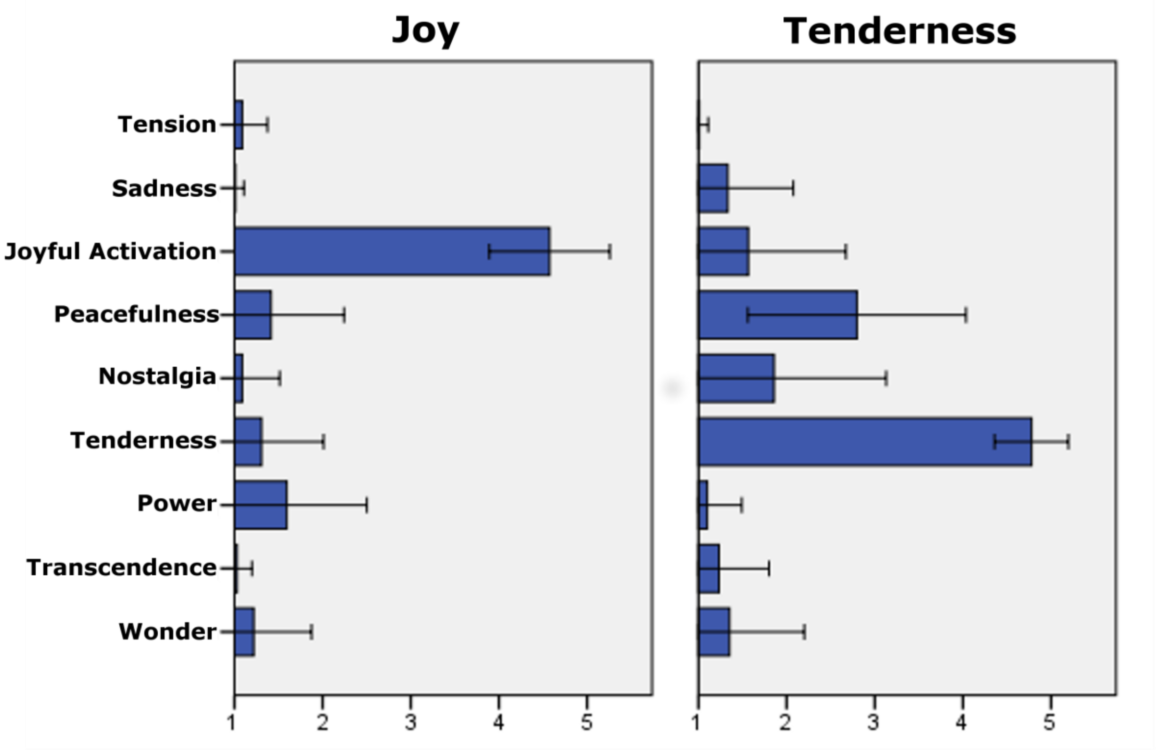


**Supplementary Figure S3. GEMS ratings of joy and tenderness evoking pieces.** Related to Methods.

Bar plots show mean and SD over 20 pieces and 6 subjects of the Genova Emotional Musical Scale (GEMS)[2]. The ratings show highest loads for the emotional target category, followed by power for joy- and peacefulness and nostalgia for tenderness-evoking pieces.

**Supplementary Table 1. Music pieces of the four medleys.** Related to Methods

| **Joyful 1** | **Piece** | **Artist** |
|---|---|---|
| *Warmup* | Fugata | Piazzola |
| 1 | Corrupião | Edu Lobo |
| 2 | Bole-Bole | Ze da Velha Silverio Pontes (Choro) |
| 3 | Mercy, Mercy, Mercy | Cannonball Adderly |
| 4 | Das Wohltemperierte Klavier-Praeludium | Bach |
| 5 | Dramophone | Caravan Palace |
| 6 | Cantaloupe Island | Herbie Hancock |
| 7 | Pret a porter de tafetá | João Bosco |
| 8 | Brandenburg Concerto No5 BWV 1050 | Bach |
| 9 | A Tapdancer's Dilema | Diablo Swing Orchestra |
| 10 | Passo Rasgado | Trio Curupira |

| **Joyful 2** | **Piece** | **Artist** |
|---|---|---|
| *Warmup* | Take Five | Dave Brubeck |
| 1 | Santa Morena | Trio Madeira Brasil |
| 2 | Zauberflöte: Papagena! Weibchen! | Mozart |
| 3 | Wolverine Blues | Thomas Finn New Orleans Ensemble |
| 4 | Andre De Sapato Novo | Altamiro Carrilho |
| 5 | Freddie Freeloader | Miles Davis |
| 6 | Desvairada | Garoto |
| 7 | Fuga y misterio | Astor Piazzolla |
| 8 | Daphne | Django Reinhardt |
| 9 | There is no Business | Irving Berlin |
| 10 | Santa Tereza | Duo Foleritmia |
| | | |
| **Tender 1** | **Piece** | **Artist** |
| *Warmup* | Sleeping in the field | Relaxmydog |
| 1 | The New World, Sym, No.9 | Dvorak |
| 2 | Porto | Renato Braz |
| 3 | Consolation No. 3 | Van Cliburn |
| 4 | Violin Concerto No1 Adagio | Bruch (Interp.Jascha Heifetz) |
| 5 | Sonata for Cello and Piano Gminor, Op.19-3 | Rachmaninov |
| 6 | Sueñoes y mentiras | Caminho |
| 7 | Fruta Boa | Milton Nascimento |
| 8 | Concerto Per Violino No 4 Rondo | Music Therapy |
| 9 | Sonata in A Minor for Arpeggione and Piano D 821 – Allegro | Schubert |
| 10 | Ballade No. 4 Op. 52 | Chopin (Rubenstein) |
| | | |
| **Tender 2** | **Piece** | **Artist** |
| *Warmup* | Prelude No1 E Minor | Andres Segovia |
| 1 | Blame it on my youth | Brad Mehldau |
| 2 | 2.Largo from Concerto for Oboe, Strings, Basso Continuo in D | Leonard Bernstein |
| 3 | Romance Op. 37 | Jean-Pierre Rampal, Marielle Nordmann |
| 4 | Meditation from Thais | Jules Massenet |
| 5 | Albert's House | Chet Baker |
| 6 | Las 4 Estaciones Portenas IV Invierno Porteno | Bragato, Munich Piano Trio |
| 7 | Dolly Op.56-1Berceuse | Fauré |
| 8 | Pavane Pour Une Infante | Ravel |
| 9 | Palhaço | Zé Paulo Becker |
| 10 | La Fille Aux Checeux | Debussy (Alexis Weissenberg) |

**References:**


1. Norman-Haignere, S., Kanwisher, N. G. & McDermott, J. H. Distinct Cortical Pathways for Music and Speech Revealed by Hypothesis-Free Voxel Decomposition. *Neuron* **88,** 1281–1296 (2015).

2. Zentner, M., Grandjean, D. & Scherer, K. R. Emotions evoked by the sound of music: characterization, classification, and measurement. *Emotion* **8,** 494–521 (2008).
